# Hesperidin efficacy of epidural fibrosis in a post laminectomy rat model: an experimental study

**DOI:** 10.55730/1300-0144.6080

**Published:** 2025-08-17

**Authors:** Emrah KESKİN, Mehmet Selim GEL

**Affiliations:** 1 Department of Neurosurgery, Zonguldak Bülent Ecevit University, Faculty of Medicine, Zonguldak, Turkiye; 2 Department of Neurosurgery, Trabzon Kanuni Training and Research Hospital, Trabzon, Turkiye

**Keywords:** Epidural fibrosis, hesperidin, antifibrotic effect, in vivo

## Abstract

**Background/aim:**

Epidural fibrosis (EF) plays a significant role in the development of failed back surgery syndrome (FBSS). This study evaluates the antifibrotic effect of hesperidin, a citrus-derived bioflavonoid, within a rat laminectomy model.

**Materials and Methods:**

Thirty-two female Wistar albino rats were randomized into four groups (n = 8 each). Group 1 received laminectomy alone (control); Group 2 received a gelatin sponge; Groups 3 and 4 intraperitoneally received 50 mg/kg and 100 mg/kg of hesperidin, respectively, for 7 days post-laminectomy. After 6 weeks, epidural tissues were assessed histopathologically, and hydroxyproline levels were measured.

**Results:**

Group 4 showed significantly lower EF grades compared with Groups 1 and 2 (p < 0.001), and reduced arachnoid invasion compared with Group 1 (p < 0.05). Group 4 had the lowest hydroxyproline levels (1.19 ± 0.05 μg/mL), followed by Group 3 (1.48 ± 0.16 μg/mL), Group 2 (1.67 ± 0.24 μg/mL), and Group 1 (1.94 ± 0.30 μg/mL) (all p < 0.001).

**Conclusion:**

Hesperidin significantly reduced EF and collagen deposition in a dose-dependent manner, demonstrating its potential as a preventive agent for FBSS.

## 1. Introduction

Failed back surgery syndrome (FBSS) is a condition that may develop post-spine surgery, wherein the patient continues to experience pain in the lower back/leg, resulting in loss of function and reduced quality of life [1]. Patients with FBSS incur serious physical, psychological, and economic losses. The etiology of FBSS is multifactorial, including preoperative factors such as inappropriate surgical indications; intraoperative factors such as inadequate surgical intervention, incorrect spinal level, and screw malposition; and postoperative factors such as disc recurrence, epidural scar tissue, and spinal stenosis [1–3]. The incidence of FBSS due to epidural fibrosis (EF) is approximately 8%–14.5% [2,3]. Alarmingly, the incidence of EF was reported to increase following reoperation to correct FBSS, causing even more distress to patients [4].

Laminectomy is an effective surgical treatment option for decompression and is widely used in spine surgery. The majority of patients undergoing laminectomy develop adhesions to varying degrees as part of the normal tissue healing response [5]. Spine surgeons are increasingly leaning toward using minimally invasive approaches because of the opinion that a small surgical field results in better healing of the paraspinal muscles and surrounding tissues (dura mater, joint, and bone), which in turn lowers the extent of adhesion formation in the epidural space [6,7]. In recent years, surgeons have favored bilateral decompression via a unilateral approach instead of instrumented surgery for treating spinal stenosis. In lumbar disc surgery, small incisions and endoscopic and interlaminar approaches are increasing in popularity in suitable patients [8,9]. The efficiency of endoscopy and microscopy plays a pivotal role in these approaches. Nevertheless, despite these advancements, EF remains one of the primary causes of FBSS. Many clinical and experimental studies have been carried out to reduce or prevent EF, demonstrating the efficacy of agents such as etanercept, curcumin, CoSeal ^®^, and pirfenidone [10–13]. However, none of these therapeutic agents have progressed to routine clinical use.

Natural compounds are often preferred in fibrosis treatment studies because of their low toxicity, easy availability, and low cost [14]. Although several bioflavonoids and natural compounds have been previously investigated in EF models, we selected hesperidin (HSP) owing to its unique combination of antioxidant, anti-inflammatory, and antifibrotic properties, all of which are highly relevant to the pathophysiology of EF formation. HSP (5,7,3′-trihydroxy-4′-methoxy-flavanone-7-rhamnoglucoside) is a flavanone glycoside bioflavonoid belonging to the flavanone group [15]. Flavonoids occur in almost all parts of a plant, including fruits, vegetables, and nuts, with HSP being the most abundant flavonoid in oranges and lemons. Since its discovery by Lebreton in 1827, several clinical and experimental studies have demonstrated its antimicrobial, antiviral, antineoplastic, anti-inflammatory, antioxidant, antifibrotic, and neuroprotective effects [15–19]. The antioxidant properties of HSP are believed to be central to all its other beneficial effects. HSP has been shown to modulate the transforming growth factor-β1/Smad and nuclear factor-κB pathways, which play key roles in fibroblast activation and collagen deposition, as demonstrated in liver and lung fibrosis models. It has been reported to alleviate oxidative stress and downregulate inflammatory cytokines such as tumor necrosis factor-α and interleukin-6 in various organ systems, including the central nervous system. However, despite its promising pharmacological profile, no study has evaluated HSP in a spinal EF model. Histopathological studies have demonstrated the active role played by HSP in preventing lung and liver fibrosis [14,18]. This experimental study investigates whether systemic administration of HSP can prevent postoperative spinal EF.

## 2. Materials and methods

This study was carried out in the Zonguldak Bülent Ecevit University Faculty of Medicine Experimental Research Center. The study’s experimental protocols w were reviewed and approved by an ethics committee (2025/02).

### 2.1. Experimental animals

This study used 32 female Wistar albino rats (age: 12–14 weeks; weight: 350–400 g). The animals were housed in ventilated cages in a controlled environment (ambient temperature: 18 °C–21 °C, atmospheric humidity: 55% ± 10%, and programmed 12-h light/dark cycles), with unrestricted access to standard rodent feed and filtered water. All animals underwent a 7-day acclimatization period prior to the experimental procedures.

### 2.2. Study design and group allocation

Rats were randomized into four experimental cohorts (n = 8 per group) using a computer-generated allocation sequence:

**Group 1 (Control)**: The rats in this group underwent standard laminectomy without additional intervention. Following hemostasis, the surgical wound was closed in anatomical layers.
**Group 2 (Gelatin matrix)**: After performing laminectomy and achieving of hemostasis, an absorbable gelatin matrix (Surgispon, Aegıs, India), 2 mm in dimension, was irrigated with 0.5 mL of sterile saline and positioned within the epidural space prior to wound closure.
**Group 3 (Low-concentration HSP)**: The rats in this group were intraperitoneally injected with HSP at 50 mg/kg body weight daily for 7 consecutive days following laminectomy.
**Group 4 (High-concentration HSP)**: The rats in this group received daily intraperitoneal injections of HSP at 100 mg/kg body weight for 7 consecutive days after laminectomy.

The dosages of HSP were selected on the basis of prior experimental studies in rats, wherein HSP demonstrated both efficacy and safety at these concentrations [20,21].

### 2.3. Surgical methodology

All surgical interventions were performed under identical conditions by the same surgical team to ensure procedural consistency. Anesthesia was established through the intraperitoneal administration of xylazine hydrochloride (5 mg/kg; Bayer) in combination with ketamine hydrochloride (25 mg/kg; Ketalar, Popular). After confirming sufficient anesthesia depth using pedal withdrawal reflex assessments, the dorsal thoracolumbar region was shaved using an electric clipper, disinfected with povidone-iodine solution, and isolated using sterile surgical drapes.

A longitudinal midline incision was made, extending from the T10 to L5 spinous processes. The thoracolumbar fascia was incised along the midline, and the paraspinal musculature was subperiosteally dissected bilaterally to expose the posterior vertebral elements spanning T12 to L4. Following clear visualization of the surgical field, total laminectomy was performed at the L1, L2, and L3 vertebral levels. The procedure included complete excision of the laminae and flavum ligaments while meticulously preserving the underlying dura mater.

Hemostasis was exclusively achieved using gentle compression with sterile cotton pledgets, deliberately avoiding electrocautery to minimize thermal injury to the neural structures. Following the primary surgical intervention, each animal received prophylactic antimicrobial therapy consisting of an intramuscular injection of cefazolin sodium (10 mg/kg; Cefamezin; Sanofi, İstanbul, Türkiye) and topical application of Neo-Caf antibiotic spray (Intervet, Italy). For the animals in Group 2, the prepared gelatin matrix was positioned directly over the exposed dura mater prior to wound closure. The animals in Groups 3 and 4 received their designated HSP regimen as described previously. Closure of the wound was performed in three distinct layers (paraspinal muscle fascia, subcutaneous tissue, and skin) using appropriate absorbable suture materials. All animals were closely monitored during recovery from anesthesia and throughout the postoperative period. No mortality or serious surgical complications were documented among the experimental animals, and all animals maintained normal mobility and neurological function throughout the study.

### 2.4. Specimen collection and processing

At 6 weeks postsurgical intervention, the animals were humanely euthanized using a standardized protocol involving the administration of pentobarbital (200 mg/kg; Penbital, Türkiye). The vertebral column was harvested en bloc from the T12 to S1 segments, carefully preserving the architecture of the epidural tissue. The specimens were meticulously dissected and appropriately segregated for histopathological and biochemical analyses. For biochemical analysis, recently obtained tissue samples were gently rinsed in physiological saline (0.9% NaCl), weighed with precision, and immediately cryopreserved at −80 °C for subsequent hydroxyproline quantification.

### 2.5. Histopathological analysis

**Figure 1:**
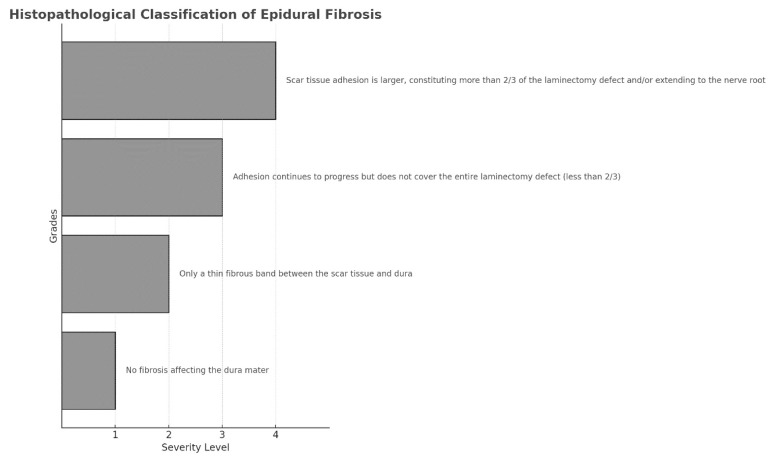
Histopathological classification of epidural fibrosis^22^.

The dissected tissues were fixed in 15% buffered formalin for 1 week and decalcified for 5 days. From each specimen, three 2-mm-thick sections were obtained from the laminectomy area and embedded in paraffin. Multiple 5-μm-thick sections were prepared axially using a rotary microtome and subjected to Masson’s trichrome staining using the Trichrome Staining Kit (Ventana Medical Systems Inc., Tucson, AZ, USA) and the BenchMark automated slide stainer (Ventana Medical Systems Inc.). The slides were then examined under the Axio Imager 2 microscope (Zeiss, Göttingen, Germany) and photographed using the Axiocam ERc 5s camera (Zeiss). To minimize assessment bias, histopathological evaluation of EF and arachnoid invasion was performed under blinded conditions by an experienced pathologist who was unaware of the group allocations. EF was evaluated according to the grading system proposed by He et al. (Figure 1) [22]. Mean values were obtained for the statistical evaluation.

### 2.6. Hydroxyproline quantification

Hydroxyproline, a specific marker for collagen deposition, was quantified using the HP Colorimetric Assay kit (Elabscience, E-BC-K062-S, USA), according to the manufacturer’s protocol. For a short duration, the tissue samples were hydrolyzed, then oxidized to produce pyrrole, which subsequently reacted with dimethylaminobenzaldehyde to generate a chromogenic product. Absorbance was measured at 550 nm, and HP concentrations were determined using a standard curve.

### 2.7. Statistical analysis

Data analysis was conducted using SPSS software, v22.0. Normality of the data was verified using the Shapiro–Wilk test, skewness/kurtosis assessment, and quantile–quantile plot examination. Hydroxyproline content and fibrosis grade, which were normally distributed variables, were compared across the four different groups using one-way analysis of variance. After identifying statistically significant differences among the groups, post-hoc analyses were conducted to identify the specific group comparisons accounting for these differences. Prior to this, the assumption of homogeneity of variances was evaluated to guide the selection of the appropriate post-hoc test. With Levene’s test showing a greater value than 0.05 and equal variances confirmed, the Scheffé post-hoc test was employed for pairwise group comparisons. Categorical variables were evaluated using likelihood ratio tests. The results are presented as mean ± standard deviation for continuous variables and as frequencies with percentages for categorical variables. Statistical significance was established at p < 0.05 throughout all analyses.

## 3. Results

### 3.1. Surgical outcomes and animal welfare

All animals survived the experimental period without any notable complications. Neurological deficits, wound infections, or adverse effects attributable to HSP administration were not detected.

### 3.2. Histopathological findings

#### 3.2.1 Epidural fibrosis grading

**Figure 2:**
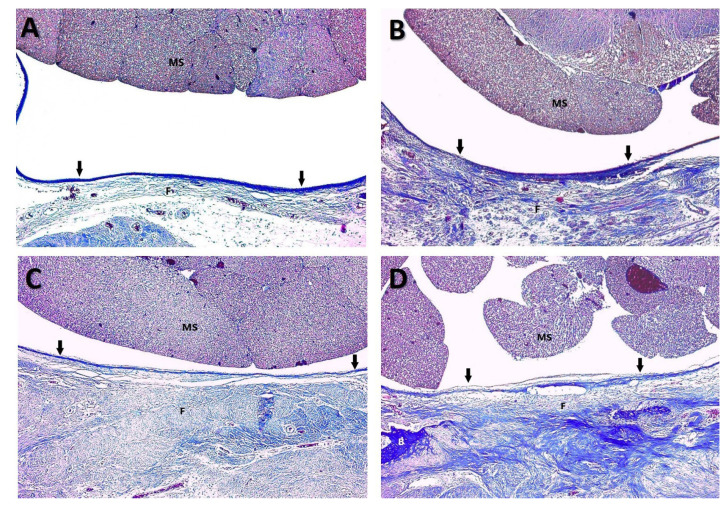
Photomicrographs of histopathologic images of groups. **A:** Grade 1 fibrosis in Group 4. Thin epidural fibrous bands (F) adhered to dura mater (arrows). (MS) Medulla spinalis. (Masson trichrome, original magnification 100x) **B:** Grade 2 fibrosis in Group 3. Epidural fibrosis (F) covered less than 2/3 of the laminectomy defect and adhered to dura matter (arrows). (MS) Medulla spinalis. (Masson trichrome, original magnification 100x) **C:** Grade 2 fibrosis in Group 2. Epidural fibrosis (F) covered less than 2/3 of the laminectomy defect and adhered to dura matter (arrows). (MS) Medulla spinalis. (Masson trichrome, original magnification 100x) **D:** Grade 3 fibrosis in Group1. Epidural fibrosis (F) completely covered the laminectomy defect and adhered to the underlying dura mater (arrows). (MS) Medulla spinalis; (B) Bone. (Masson trichrome, original magnification 100x).

Histological examination revealed significant between-group differences in EF severity (p ≤ 0.001). The high-dose HSP group demonstrated markedly reduced fibrosis compared with both the control and gelatin sponge groups (p ≤ 0.001 for both comparisons; Table 1; Figures 2A, B, C, D). No significant difference in EF severity was observed between the low-dose HSP group and the gelatin sponge group (Table 1; Figures 2B, C).

Grade 3 fibrosis was predominantly observed in the control group (75% of specimens) and the gelatin sponge group (62.5% of specimens), whereas it was entirely absent in the high-dose HSP group. The distribution of fibrosis grades across the experimental groups is summarized in Table 2.

#### 3.2.2. Arachnoid invasion

Arachnoid membrane invasion by fibrotic tissue demonstrated significant between-group variation (p < 0.05, Table 3). Notably, arachnoid invasion was completely absent in the high-dose HSP group, whereas its incidence in the control, gelatin sponge, and low-dose HSP groups was 62.5%, 50%, and 25%, respectively (Table 3).

#### 3.2.3. Histomorphological characteristics

Histomorphological analysis revealed distinct differences in epidural tissue architecture between the treatment groups. The control and gelatin sponge groups exhibited dense, disorganized collagen deposition with abundant fibroblast infiltration and neovascularization in the epidural space. By contrast, the high-dose HSP group demonstrated markedly reduced collagen density with minimal fibroblast proliferation and preservation of the epidural space architecture.

### 3.3. Hydroxyproline quantification

**Figure 3:**
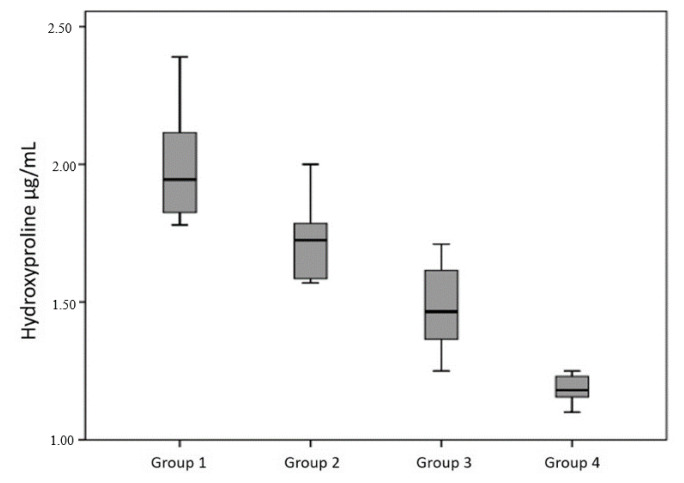
Hydroxyproline level (μg/mL) expressed as the mean ± standard deviation of wet tissue. The high dose hesperidin group showed the lowest level.

The hydroxyproline content of tissues exhibited significant between-group differences (p < 0.001, Table 4). The mean hydroxyproline concentration progressively decreased across the experimental groups, with the highest levels observed in the control group (1.94 ± 0.30 μg/mL) and the lowest in the high-dose HSP group (1.19 ± 0.05 μg/mL; Figure 3).

Posthoc analysis demonstrated that hydroxyproline levels in the high-dose HSP group were significantly lower than those in the control, gelatin sponge, and low-dose HSP groups (all p < 0.001, Table 4). Additionally, the low-dose HSP group demonstrated significantly reduced hydroxyproline content compared with the control group (p < 0.05, Table 4).

## 4. Discussion

Failed back surgery syndrome is a complex and multifactorial problem in which surgical success could not be achieved after back surgery. One of the most prevalent causes of FBSS is EF, which is characterized by the development of scar tissue in the epidural space [5, 23]. Various preclinical and clinical studies have evaluated the efficacy of various therapeutic agents in preventing the development of EF after lumbar spine surgery [24–26]. A study conducted on rats demonstrated the potential of boric acid to reduce the development of EF after spinal surgery [24]. Another study showed that ranibizumab, a vascular endothelial growth factor inhibitor, attenuated the development of EF in a post laminectomy rat model [25].

However, not all studies investigating the efficacy of therapeutics in preventing EF have yielded positive results. In a study using a post laminectomy rat model, Dinc et al. did not observe any difference in EF development between control rats and rifamycin-treated rats [27]. The effect of topical curcumin application on EF development was examined using a similar model, but no statistically significant results were obtained [28].

Studies have suggested that myofibroblast differentiation could be related to the development of EF. Jiang et al. demonstrated that the serum response factor/myocardin-related transcription factor-A signaling pathway plays a key role in regulating myofibroblast differentiation in the development of EF [29]. In their in vitro study, Yang et al. found that suberoxylanilide hydroxamic acid reduced EF by inhibiting myofibroblast differentiation [26]. Wu et al. demonstrated the inhibitory effects of HSP on in vitro myofibroblast activity [30].

HSP is known to exert antifibrotic effects through several interconnected molecular pathways. One of the key mechanisms involves inhibition of the transforming growth factor-β1/Smad signaling pathway, which plays a central role in fibroblast activation, collagen synthesis, and extracellular matrix deposition—all critical steps in fibrosis development [18]. By downregulating the expression of transforming growth factor-β1 and its downstream mediators (e.g., Smad2/3 phosphorylation), HSP may attenuate fibroblast-to-myofibroblast differentiation and reduce collagen accumulation. Moreover, HSP produces antioxidant and anti-inflammatory effects by suppressing nuclear factor-κB activation and reducing the levels of proinflammatory cytokines, such as tumor necrosis factor-α and interleukin-1β. These molecular effects likely limit the inflammatory response following tissue injury, a known driver of fibrotic tissue remodeling in the epidural space. Experimental studies also suggest that HSP modulates oxidative stress pathways, including the nuclear factor erythroid 2–related factor 2/heme oxygenase-1 axis, thereby protecting the neural and surrounding connective tissue from damage due to reactive oxygen species, which is a major contributor to chronic inflammation and fibrosis [31]. Finally, HSP has been shown to inhibit angiogenesis and fibroblast proliferation, which may further contribute to the preservation of normal epidural architecture and the prevention of disorganized scar formation [32,33].

HSP is a citrus fruit flavone that has been shown to exert antifibrotic, antioxidant, neuroprotective, antiinflammatory, antineoplastic, antidiabetic, and cardio modulatory effects on different tissues [32–41]. The antiinflammatory effects of HSP are likely mediated by the nuclear factor-κB signaling pathway [34]. Hajialyani et al. reviewed the neuroprotective activity of HSP, highlighting its inhibitory effect on the progression of neurodegenerative diseases such as Parkinson’s disease, Alzheimer’s disease, Huntington’s disease and multiple sclerosis [35]. Another review of the health-promoting effects of HSP elaborated on its anti-inflammatory, insulin-sensitizing, antioxidant, and lipid-lowering effects [36]. Aggarwal et al. showed that in cancer cells, HSP inhibits cell proliferation, triggers apoptosis, arrests the cell cycle, inhibits angiogenesis, prevents tumor cell metastasis, and increases chemosensitivity [37]. A review by Ferreira de Oliviera et al. focused on the ability of HSP to modulate cell cycle regulation and trigger apoptosis [40]. Some studies have also explored the effects of HSP on hunger and diabetes. Suzuki et al. reviewed the effect of HSP on ghrelin signaling, highlighting its orexigenic and prokinetic activities. The authors concluded that HSP had a wide range of effects on biological functions, including inducing apoptosis, suppressing cancer cell proliferation, and promoting ghrelin secretion in the stomach via serotonin receptor antagonism [38].

Another prominent area of HSP research involves its effects on cardiovascular risk factors. In a review, Mas-Capdevila et al. stated that HSP reduces blood glucose levels, one of the most prominent cardiovascular risk factors. Moreover, they described the anti-inflammatory effects of HSP in patients with diabetes and its antihypertensive and antioxidant effects in patients with hypertension [42]. In a double-blind randomized controlled study focused on metabolic syndrome, Yari et al. reported that the HSP group exhibited lower levels of serum triglycerides, total cholesterol, low-density-lipoprotein cholesterol, tumor necrosis factor-α, and high-sensitive C-reactive protein, compared with the control group [43]. However, some studies have reported nonpositive or neutral effects of HSP. Shams-Rad et al. conducted a meta-analysis on the effects of HSP on fasting blood glucose levels, encompassing 318 participants over six studies. Their results indicated that HSP did not exert a significant effect on fasting blood glucose levels [44]. Another study investigated the effect of HSP on the performance of amateur cyclists. At the end of the 8-week experimental period, functional threshold power and maximum power levels were elevated in the HSP group compared with the control group, but changes in the anaerobic ventilatory threshold and oxygen volume were nonsignificant between the two groups [45].

Histopathological investigations of the effects of HSP have previously been performed in studies on spinal cord injuries [46]. In addition, hydroxyproline has been used as an indicator of fibrosis in previous studies [46,47]. In our study, we employed tissue histopathological evaluations and hydroxyproline quantification to assess the antifibrotic effects of HSP. To our knowledge, this is the first study to investigate the effects of HSP on the development of EF, and its findings of this will serve as a foundation for future clinical and larger-cohort studies on EF. HSP has already been approved for clinical use in other fields, and with further validating research, it may be developed for clinical use in the management of EF [48–51].

Although our findings provide the first preclinical evidence for the antifibrotic potential of systemic HSP administration in preventing EF, the translational relevance of these results to human clinical practice should be interpreted with caution. Although rat models are valuable for controlled experimental evaluation, they may not fully replicate the complexity of human spinal wound healing, immunological responses, and scar formation. Species-specific anatomical and physiological differences can influence drug metabolism, bioavailability, and fibrotic processes. Moreover, our study was limited to histological and biochemical endpoints without functional or behavioral assessments that would better reflect the clinical outcomes. Therefore, before HSP can be considered for clinical use in the prevention of FBSS, further research is warranted involving larger animal models, longer follow-up periods, mechanistic studies, and ultimately, well-designed human clinical trials.

## 5. Study limitations

This study has some noteworthy limitations. First, the sample size was relatively limited (n = 8 per group), and a formal power analysis was not conducted, which may constrain the statistical reliability of the findings. Second, the follow-up period was limited to 6 weeks. Although this timeframe is in line with similar experimental studies, longer durations might better reflect the progression and recurrence of chronic fibrosis. Third, the study did not include functional or behavioral assessments, which would have provided additional insight into the clinical relevance of the histological findings. Furthermore, the lack of mechanistic investigations, such as immunohistochemical staining for fibroblast activity, collagen subtypes, or inflammatory cytokines, precludes a deeper understanding of the cellular pathways through which HSP may exert its antifibrotic effects. Finally, the absence of a sham-operated group hinders the ability to distinguish between the effects of surgical trauma and those resulting specifically from the laminectomy.

## 6. Conclusion

The flavonoid HSP has been shown to exert antioxidant, antifibrotic, antineoplastic, and anti-inflammatory effects in different tissues. According to our findings, HSP is a promising natural therapeutic for alleviating the development of FBSS in post laminectomy patients.

## Figures and Tables

**Table 1:** Differences between groups according to fibrosis grades.

Groups	n	χ̄ ±s d	Differences groups	p*
1	8	2.75 ± 0.46	1–4	
2	8	2.63 ± 0.52	2–4	≤0.001*
3	8	2.00 ± 0.76	-	
4	8	1.63 ± 0.52	4–1, 4–2	

One way Anova with Scheffe test was used for differences among the groups

p* was shown as a significant difference at the 0.05 level; Group 1: Control, Group 2: Gelatin matrix, Group 3: Low dose hesperidin, Group 4: High dose hesperidin

**Table 2:** Fibrosis grades of groups.

Groups n: 8	Grade 1	Grade 2	Grade 3
**1**		2(25%)	6(75%)
**2**		3(37.5%)	5(62.5%)
**3**	2(25%)	4(50%)	2(25%)
**4**	3(37.5%)	5(62.5%)	

Group 1: Control, Group 2: Gelatin matrix, Group 3: Low dose hesperidin, Group 4: High dose hesperidin

**Table 3:** Differences Between Groups according to Arachnoidal involvement.

Groups	(−)	(+)	p*
**1**	3(37.5%)	5(62.5%)	<0.05*
**2**	4(50%)	4(50%)	
**3**	6(75%)	2(25%)	
**4**	8(100%)	-	

* Likelihood ratio test, (−) no arachnoidal involvement, (+) arachnoidal involvement, Group 1: Control, Group 2: Gelatin matrix, Group 3: Low dose hesperidin, Group 4: High dose hesperidin

**Table 4:** Differences between groups according to Hydroxyproline.

Groups	N	χ̄ ± sd (μg/mL)	Differences groups	p*
1	8	1.94 ± 0.30	1–2, 1–4	
2	8	1.67 ± 0.24	2–4	<0.001*
3	8	1.48 ± 0.16	3–1	
4	8	1.19 ± 0.05	4–1, 4–2	

One Way Anova with Scheffe posthoc test was used for differences among the groups

p* was shown as a significant difference at the 0.05 level; Group 1: Control, Group 2: Gelatin matrix, Group 3: Low dose hesperidin, Group 4: High dose hesperidin
